# The P Value Line Dance: When Does the Music Stop?

**DOI:** 10.2196/21345

**Published:** 2020-08-27

**Authors:** Marcus Bendtsen

**Affiliations:** 1 Department of Health, Medicine and Caring Sciences Division of Society and Health Linköping Sweden

**Keywords:** sample size, randomized controlled trial, Bayesian analysis, *P* value, dichotomization, dichotomy, error, uncertainty

## Abstract

When should a trial stop? Such a seemingly innocent question evokes concerns of type I and II errors among those who believe that certainty can be the product of uncertainty and among researchers who have been told that they need to carefully calculate sample sizes, consider multiplicity, and not spend *P* values on interim analyses. However, the endeavor to dichotomize evidence into significant and nonsignificant has led to the basic driving force of science, namely uncertainty, to take a back seat. In this viewpoint we discuss that if testing the null hypothesis is the ultimate goal of science, then we need not worry about writing protocols, consider ethics, apply for funding, or run any experiments at all—all null hypotheses will be rejected at some point—everything has an effect. The job of science should be to unearth the uncertainties of the effects of treatments, not to test their difference from zero. We also show the fickleness of *P* values, how they may one day point to statistically significant results; and after a few more participants have been recruited, the once statistically significant effect suddenly disappears. We show plots which we hope would intuitively highlight that all assessments of evidence will fluctuate over time. Finally, we discuss the remedy in the form of Bayesian methods, where uncertainty leads; and which allows for continuous decision making to stop or continue recruitment, as new data from a trial is accumulated.

## Introduction

The (ab-)use of *P* values—the great divider of evidence, minds, and hearts—is, despite a great deal of critique [1-4], still going strong. It is remarkable that less than 60 years ago Hill wrote: “Fortunately I believe we have not yet gone so far as our friends in the USA where, I am told, some editors of journals will return an article because tests of significance have not been applied” [5]. The pendulum has unfortunately swung, as statistical significance has become the arbiter in many scientific disciplines, taking precedence over real-world impact of results, model critique, data quality, etc.

But is it not of the upmost importance to science to have a method to decide if an intervention has an effect? The answer is, to some rather surprisingly, a resounding No. There is no need to spend endless hours writing grant applications, thoughtfully designing experiments, tirelessly recruiting participants, and then chasing follow-up data to reduce attrition—if all you want to know is if an intervention has an effect, then the answer is Yes - all interventions have an effect and you can prove it using *P* value dichotomization as long as you have enough data [6]. The smaller effect you wish to identify, the larger the required sample size will be [7]; and at some point, the sample size required may be greater than the population at hand, which makes the experiment impossible. However, the null hypothesis will always fall given enough data.

This viewpoint will present 2 real-world examples, which will hopefully convince the reader that *P* value dichotomization is not helping scientific discovery and that the praxis needs to be reconsidered. The two trials discussed in this viewpoint have received ethical approval: Regional Ethical Committee in Linköping, Sweden (Dnr 2017/388-31 and Dnr 2018/417-31).

## If we Could Only Recruit Some More People, This Smoking Cessation Intervention Would Become Effective!

In our first example, we will look back at an experiment conducted among high school students in Sweden [8,9]. The effects of a text messaging intervention on smoking cessation was being estimated, in comparison to a waiting list control group. A 2-arm single blind trial design was used, with equal probability randomization to both groups.

There were 2 outcome measures: prolonged abstinence of smoking (not smoking more than 5 cigarettes over the past 8 weeks) and point prevalence of smoking cessation (not smoking any cigarette the past 4 weeks). Findings suggested, 3 months after randomization, that the effect of the intervention on prolonged abstinence could not be distinguished from the null (OR 1.25, 95% CI 0.78-2.01; *P*=.36); however, the effect on point prevalence could be (OR 1.83, 95% CI 1.12-3.02; *P*=.017).

Recruitment was initially planned to last for 6 months [9]. However, after this time had elapsed, only 386 students had been recruited, less than the prespecified goal of 558. Therefore, it was decided to extend the recruitment period by another 6 months, after which recruitment stopped; and a total of 535 students were recruited.

**Figure 1:**
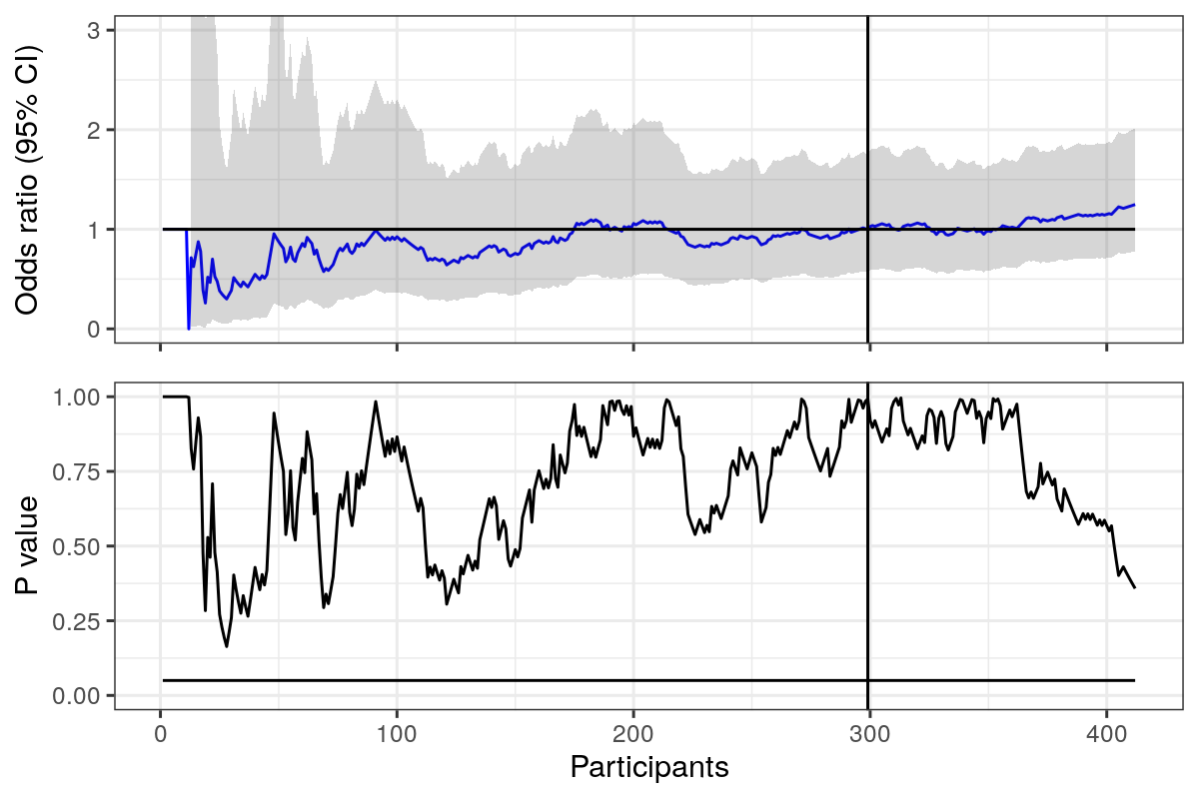
Prolonged abstinence: odds ratios and *P* values calculated using actual data from trial, plotted over time (number of responders in the study). Horizontal lines represent null value (OR 1) and the .05 statistical significance line. Vertical line represents where the first 6 months of recruitment ended.

**Figure 2:**
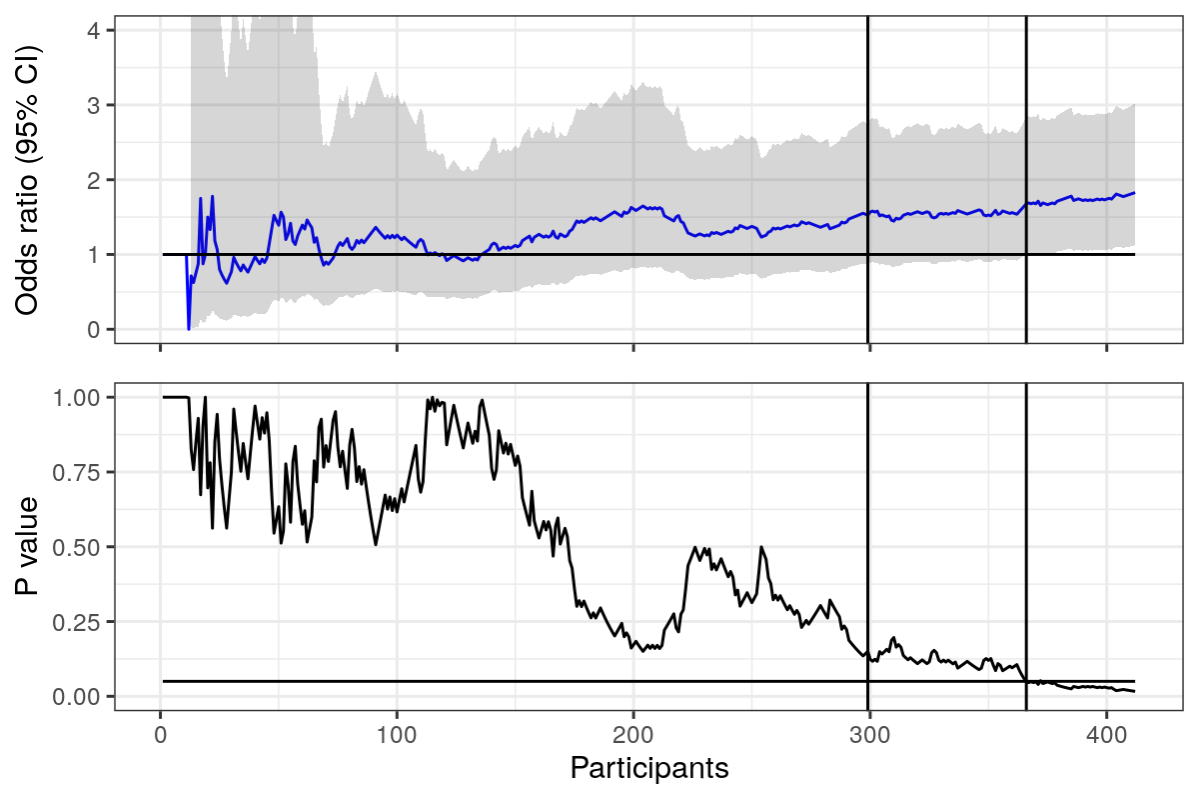
Point prevalence: odds ratios and *P* values calculated using actual data from trial, plotted over time (number of responders in the study). Horizontal lines represent null value (OR 1) and the .05 statistical significance line. First vertical line represents where the first 6 months of recruitment ended, second vertical line represents when point prevalence became statistically significant.

What would our null hypothesis–focused analyses have looked like had we decided to stop after recruiting 2 participants? 4? 50? 400? In Figure 1 and Figure 2 we have drawn plots, which represent our analyses of prolonged abstinence (Figure 1) and point prevalence (Figure 2) given a certain number of responders. Follow-up attrition was relatively high in this trial, however, for now we will use responders and participants interchangeably.

Looking at Figure 1, we can see that odds ratios for prolonged abstinence fluctuate heavily when there are few responders, but then seem to settle a bit as the number increases. We should expect this from point estimates, as when there are few data, each point plays a larger role in the estimate. The *P* values are highly unstable, fluctuating even when the number of responders is large, but never crossing the magic .05 significance line. The vertical line represents the 6-month mark, when the trial was initially planned to stop recruitment. Looking only at the *P* value, our conclusions would not have been much different had we decided to stop at this point. Since it was not significant, the OR is irrelevant (or is it?). However, the estimated odds ratios were different after 6 and 12 months (OR 1.00 vs OR 1.21).

Focusing instead on Figure 2, where point prevalence is analyzed, we see a similar story early on, ORs and *P* values fluctuate, but then things seem to settle a bit. Had the trial ended at the 6-month mark (the left most vertical line), we would have concluded that the effects of the intervention were not distinguishable from the null, thus not rejected the null hypothesis. However, at the end of 12 months, we can see that the *P* value was below the .05 significance line, suggesting statistical significance and that the effect of the intervention was distinguishable from the null. Finally! After respondent 366 (right most vertical line), we can look at our OR in new light - the OR of 1.68 is statistically significant, there is an effect! Sadly, had we stopped at the previous respondent (number 365), the OR of 1.64 would not have been significant, indistinguishable from the null and not a measure which we should interpret as an effect of the intervention.

**Figure 3:**
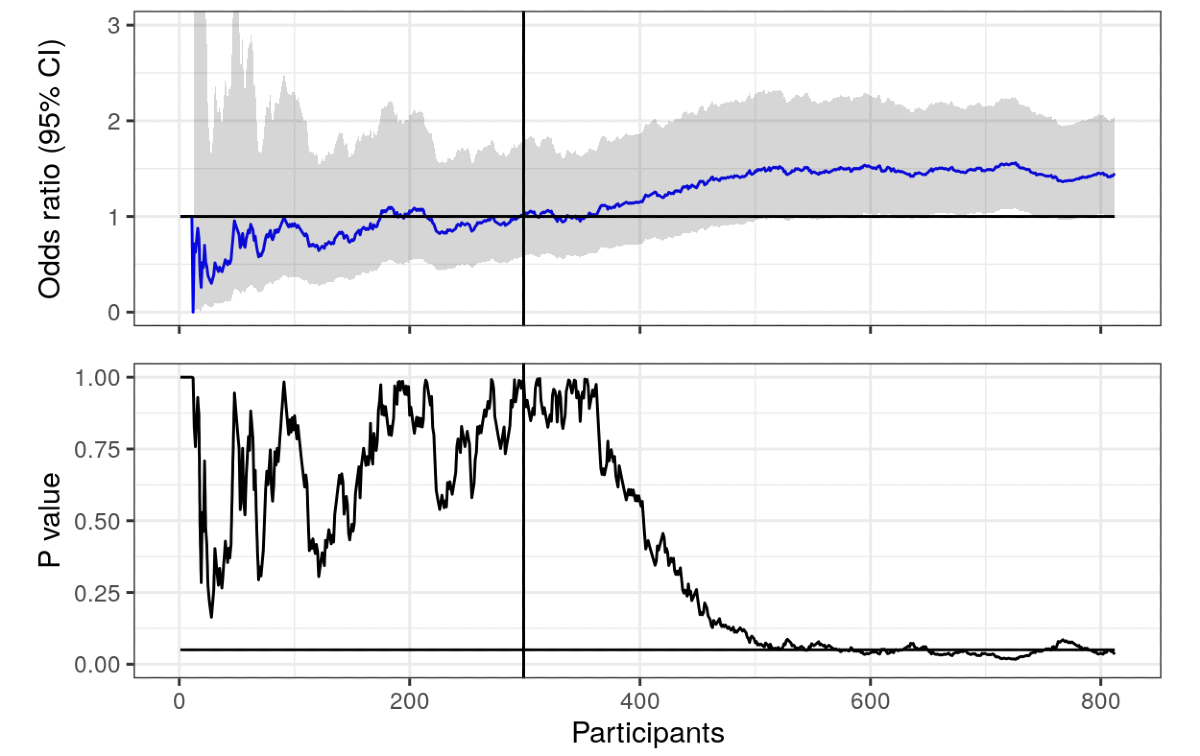
Prolonged abstinence (with sampled data): odds ratios and *P* values calculated using actual and sampled data from trial, plotted over time (number of responders in the study). Horizontal lines represent null value (OR 1) and the .05 statistical significance line. The vertical line represents where the first 6 months of recruitment ended.

**Figure 4:**
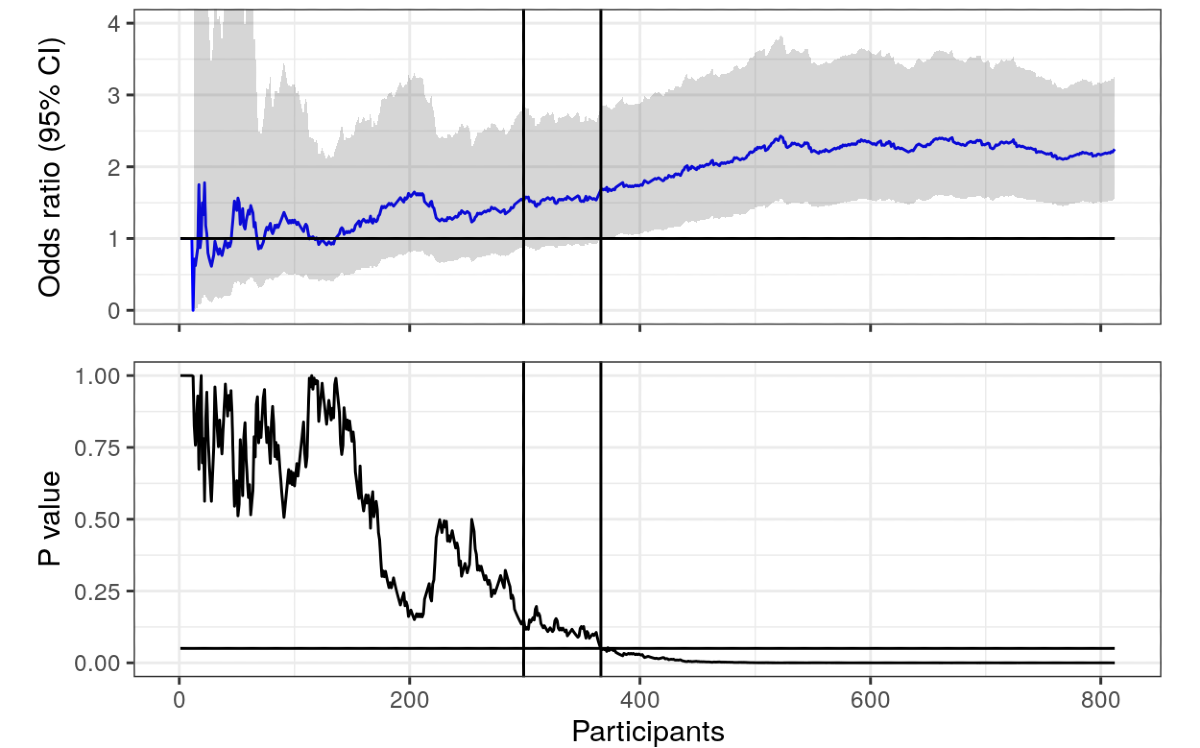
Point prevalence (with sampled data): odds ratios and *P* values calculated using actual and sampled data from trial, plotted over time (number of responders in the study). Horizontal lines represent null value (OR 1) and the .05 statistical significance line. First vertical line represents where the first 6 months of recruitment ended, second vertical line represents when point prevalence became statistically significant.

What if we had continued recruitment? What if we had another 400 students take part in our trial? Well, we cannot possibly know exactly how these students would have responded; but for the sake of this experiment, it is not strictly necessary. We can pretend that the new 400 participants are similar to the participants we already have, and therefore, sample 400 respondents from those already in our trial (with replacement). The new OR and *P* value timeline can be seen in Figure 3 and Figure 4.

As we can see in Figure 3, it turns out that the intervention actually did have an effect on prolonged abstinence. We just did not have enough respondents to distinguish it from the null using the .05 threshold, but now we do. We could argue that resampling from a sample with a nontrivial OR and recalculating the *P* value will of course result in a lower *P* value, but that is exactly the point! Statistical significance is a function of the sample size, so any effect can be statistically significant if there are enough participants; and all interventions have an effect [6,7]. Note that there is a lot of crossing the significance line between 600 and 800 respondents, many opportunities to end the trial and cry wolf. In Figure 4, the *P* value has essentially flatlined.

So, should we simply continue recruiting until our *P* values flatline, forgoing a prespecified sample size? That is likely a bad idea if there are harms and costs involved, which would make it ethically irresponsible. One may argue that if you cannot reject the null given your prespecified effect and sample size, then the value of flatlining the *P* value at some smaller effect size is not worth the risk. However, such thinking may be irresponsible, as we shall see.

## But it was Statistically Significant Yesterday!

Our second example concerns an experiment of estimating the effects of receiving 1 of 2 different text messages with alcohol and health information. This experiment was nested within a larger (ongoing) trial of a digital alcohol intervention [10]. Participants who were randomized to the control arm of the trial were randomized further into 2 arms. The first arm received a public health message regarding alcohol, violence, and cancer. The second arm also received information about alcohol, violence, and cancer, but the information was worded in an alcohol industry manner, focusing on responsible drinking and downplaying the evidence on the risks of alcohol. At the end of both text messages was a hyperlink, which lead to more information about alcohol and health, and the experiment outcome was whether or not participants pressed the hyperlink.

**Figure 5:**
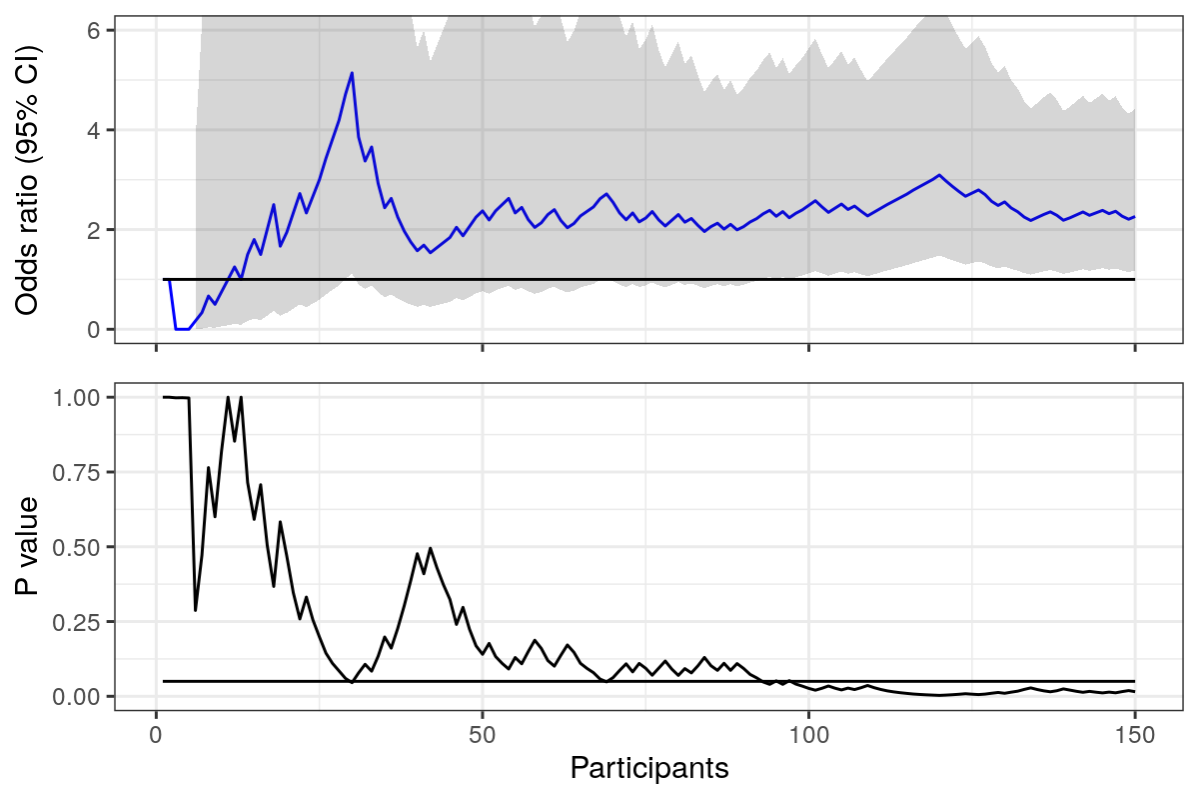
Pressed link in text message: odds ratios and *P* values calculated and plotted over time (number of participants in the study). Horizontal lines represent null value (OR 1) and the .05 statistical significance line.

After having recruited 150 participants in the experiment, we were curious to see how things were progressing. Interim analyses were interesting. It turned out that participants in the public health arm were far more likely to press the link (OR 2.26, 95% CI 1.18-4.42; *P*=.015). Figure 5 shows, as before, ORs and *P* values given a certain number of participants. The *P* value does at first glance look like it has flatlined.

**Figure 6:**
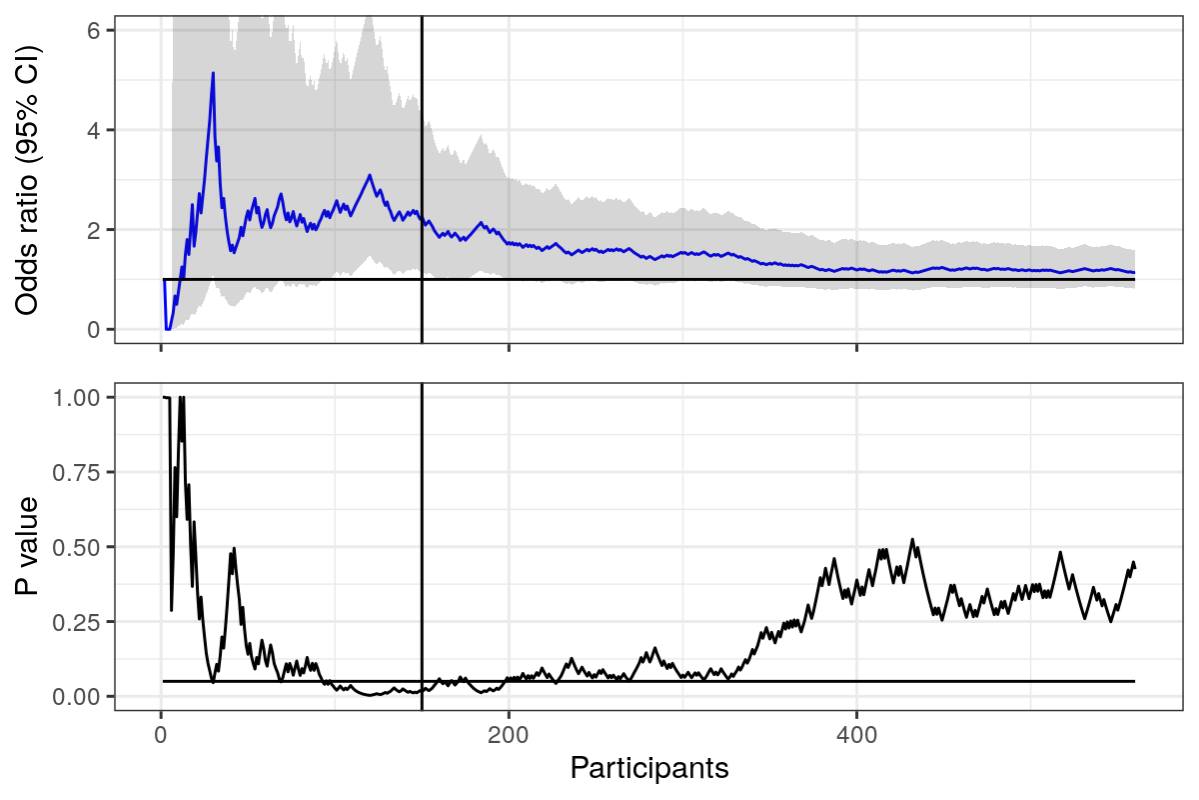
Pressed link in text message: odds ratios and *P* values calculated and plotted over time (number of participants in the study). Horizontal lines represent null value (OR 1) and the .05 statistical significance line. Vertical line represents when interim analyses were conducted.

However, as the trial progressed, and more participants were recruited, the fickleness [11] of the *P* value became apparent. After one year, 560 participants had been recruited, and the ORs and *P* values plot (Figure 6) looked markedly different. Now, there was no statistical significance (OR 1.14, 95% CI 0.82-1.59; *P*=.43). However, there were plenty of times where the trial might have ended, and a statistically significant result would have been the result; and if the trial continues recruitment, we will eventually have a significant result again (as discussed in the previous example).

There was no prespecified sample size for this exploratory outcome, since it was nested within a larger trial. However, think about the number of times you have read reports of trials, and grant and ethics applications, where the power analysis has concluded that recruiting approximately 150 participants will suffice. Given a large enough effect size, this will hold for the calculation; and if researchers are “lucky,” it will hold in their experiment. How many reports have you read where statistically significant results have led to a discussion about important results based on 150 participants? What would have happened if they continued recruiting another 50, 100, or 200 participants?

## Letting Bayes be the Conductor

One of the core issues underlying the 2 examples given herein is that point estimates and *P* values are very fickle when taking the traditional approach. This fickleness is caused by assuming that data alone is all we care about, and we take no action towards tempering our expectations. That is, after collecting data from 2 participants, 1 from the intervention group and 1 from the control group, the effect is estimated to be the difference between these two. Put in another way, we are susceptible to drawing conclusions from small sample sizes, and we ignore our belief that our interventions will likely have small to modest effects.

An alternative is to take a Bayesian approach to inference [12-14]. While a full discussion about the details of Bayesian inference cannot possibly fit here, the essentials can be captured as follows: You count what you see (the data), and balance this with what you expect (known as the *prior*). For instance, if you believe that there may be both black and white swans (this is your prior), then you do not make the conclusion that all swans are white after having seen a single white swan. After having seen thousands of white swans you may decide that it is more likely that swans are white, but you do not say that it is impossible for swans to be black.

From a Bayesian perspective, a trial is a series of repeated experiments; and each time we collect data from a participant, we can update our inference about the trial outcomes. This is often referred to as a Bayesian group sequential design [15,16]. We use prespecified criteria to decide if a trial is a success, or if it is futile to continue recruitment (and possibly also if it is unethical [17] to continue due to harm). These criteria can be evaluated as many times as we like. Using our second example from before, where we studied prevalence of pressing on a link in a text message, we may define our success criteria as: *If there is more than a 95% probability that the OR is greater than 1, we end the trial and call it a success*. A criterion for futility may be as follows: *If there is more than a 95% probability that the OR is somewhere between 1/1.25 and 1.25*, *then we believe that the groups are essentially equal, and there is no need to further investigate the intervention*. These 2 probabilities (for success and futility) can then be calculated using what is commonly referred to as a *skeptical* prior, which encodes a strong a priori belief that the intervention has no effect, and that the data needs to convince us otherwise.

**Figure 7:**
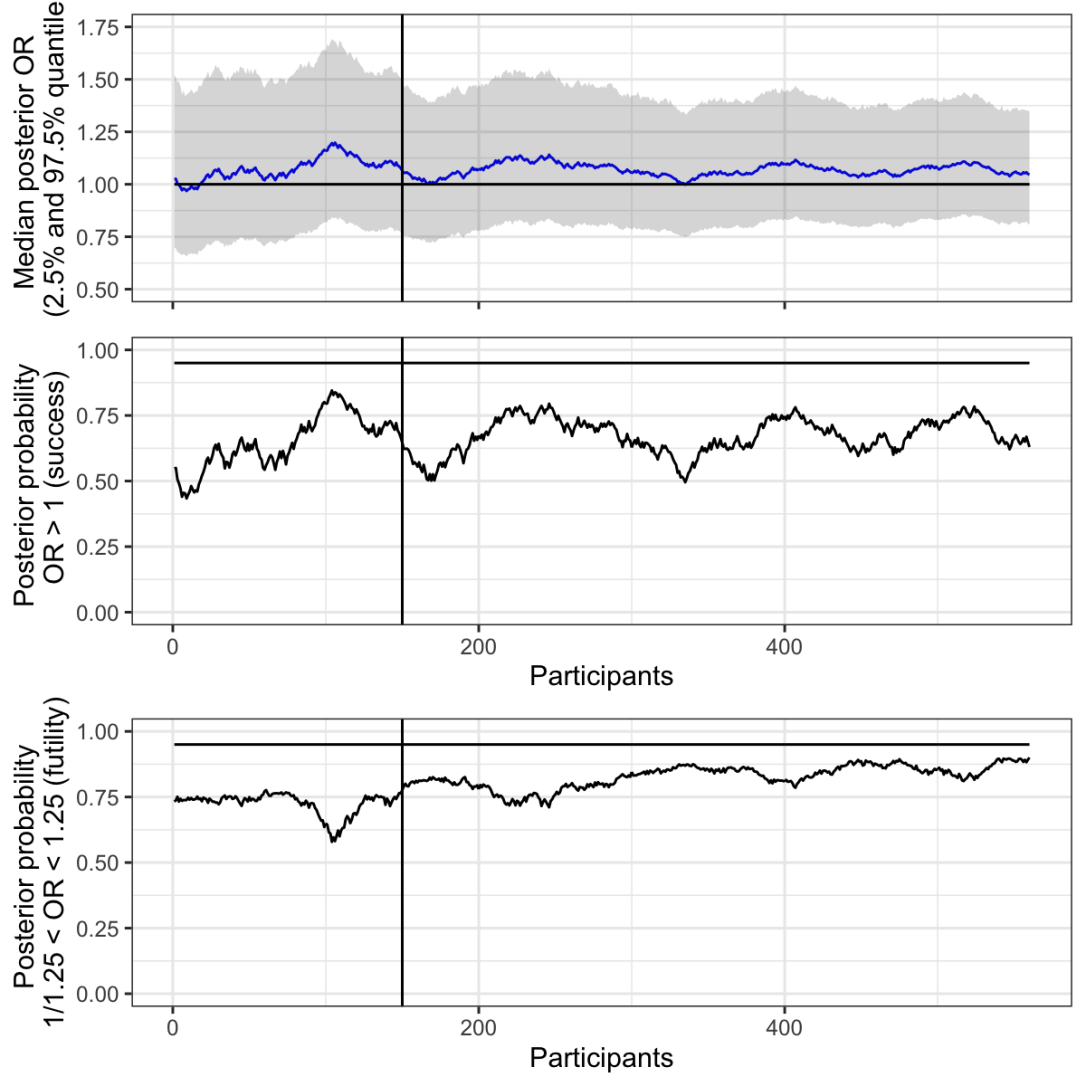
Pressed link in text message: median posterior odds ratios and probability of success and futility plotted over time (number of participants in study). Horizontal line represents null value (OR 1), and 95% probability of success and futility respectively. Vertical line represents interim analysis. A skeptical prior (mean 0, SD 0.2) was used for all inference.

In Figure 7, we have plotted the median OR (top plot), the probability of success (middle plot), and the probability of futility (lower plot) given the criteria set above. A skeptical prior for the intervention effect (normal distribution with mean 0 and SD 0.2) has been used for both the success and futility criteria. While full details are scarce in this viewpoint, it should be clear from these plots that by tempering our expectations, we have avoided making conclusions early. The *P* value approach (Figure 6) called for a statistically significant effect after 150 participants (vertical line), which was later overturned. We have avoided this by using a Bayesian approach; and it looks like the experiment will be considered futile as new data is collected, as the probability of an OR between 1/1.25 and 1.25 (lower plot) is gradually increasing towards 95% (our predefined futility criteria).

## Discussion

The 2 examples herein are not fictive, and they are by no means unique. We invite readers to plot effect estimates and *P* values in a similar fashion as we have and reflect on the robustness of their past conclusions. If such plots were commonplace in scientific papers, would readers' or reviewers' interpretations of the findings change? There is no finger pointing here, we are all as a collective responsible to ensure that the scientific method is sound.

The replication crisis in the social sciences is proof that methods built on dichotomization of evidence are not scientific [18]. It needs to stop. However, if researchers, journals, reviewers, funding agencies, media, and the general public, continue to crave statements of true and false—effect or no effect—then there is no silver bullet, which will make the line dancing cease [19]. Consulting confidence intervals is veiled hypothesis testing, and reducing the *P* value threshold to .005 [20] is just kicking the can down the road and opening the door for new *P* value–hacking and selective reporting issues [1]. Add to this that *P* values (and confidence intervals) are consistently being misinterpreted [4,21], and even highly respected journals are allowing nonsignificance to be interpreted as an absence of effect [22]. A recent example was the conclusion that lopinavir–ritonavir treatment for COVID-19 “was not beneficial in comparison to standard care*”* [23], backed by a hazard ratio for clinical improvement of 1.31 with a 95% CI 0.95-1.80, which is not statistically significant, but which cannot rule out a hazard ratio of 1.80.

What is the alternative? Well, as Gelman and Carlin put it [19], “...resist giving clean answers when that is not warranted by the data. Instead, do the work to present statistical conclusions with uncertainty rather than as dichotomies.” Doing so is natural from a Bayesian perspective, as posterior distributions can directly describe the relative compatibility of different models given the data (rather than the other way around). In fact, Bayesian inference answers the question that researchers want to ask (but have been told that they cannot): What is the probability that an intervention had a positive effect? Interventions should not be dismissed because the design of an experiment did not allow the *P* value line to be crossed, as we have seen, it may be sheer luck that an experiment stopped exactly when it could show significance.

We recognize the importance for careful planning of trials, including giving estimates on the number of individuals necessary to recruit. However, prespecifying sample sizes based on type I and type II errors is not only ignorant to the fact that it is not possible to know how many individuals are necessary to recruit (it may be considered a random variable itself), but may also be considered unethical as it may lead to over-recruitment, detecting harm and benefit later than necessary [16]. Using multiple looks at the data throughout the trial and making judgments based on null hypothesis tests is not only problematic due to its reliance on fickle point estimates (as demonstrated herein), but also inflates type I errors due to multiplicity if not handled correctly [24]. Instead, a Bayesian group sequential design [15,16] allows for continuous monitoring as data is collected, utilizing target posterior probabilities for success and futility, such that a decision can be made to stop or continue recruitment each time new data is available without concern for multiplicity [25].

It should be noted that all assessments of evidence will fluctuate over time, as we have shown in the enclosed examples. One aspect of this is that smaller samples may not represent the study population well. Another is that changes may occur in the underlying study population as we recruit over time, which means that we may be sampling from different regimes [26-28] in the data (for instance, due to seasonal differences). However, in a Bayesian group sequential design we can use a skeptical prior which will draw back the posterior probability of effect and posterior median, which will automatically correct for too early looks at the data [25]. This goes some way towards protecting from small sample sizes and regimes that may be misrepresentative of the population we wish to study.

Uncertainty is the driving force of science, and uncertainty *in* can never result in certainty *out*. Uncertainty leads in Bayesian methods, and it allows us to more clearly judge our findings in light of it. Convoluted rules for sample size estimation, *P* value spending, or correction for multiplicity are all artifacts from thinking that certainty can be the result of uncertainty. We can increase our understanding of the uncertain through repeating experiments, as was Fisher's intention, which ultimately is the goal of science [29].
